# Systematic Regional Variations in Purkinje Cell Spiking Patterns

**DOI:** 10.1371/journal.pone.0105633

**Published:** 2014-08-21

**Authors:** Jianqiang Xiao, Nadia L. Cerminara, Yuriy Kotsurovskyy, Hanako Aoki, Amelia Burroughs, Andrew K. Wise, Yuanjun Luo, Sarah P. Marshall, Izumi Sugihara, Richard Apps, Eric J. Lang

**Affiliations:** 1 Department of Neuroscience & Physiology, New York University School of Medicine, New York, New York, United States of America; 2 School of Physiology and Pharmacology, University of Bristol, Bristol, United Kingdom; 3 The Bionics Institute, East Melbourne, Victoria, Australia; 4 Department of Systems Neurophysiology, Graduate School of Medicine, Tokyo Medical and Dental University, Tokyo, Japan; 5 Center for Brain Integration Research, Tokyo Medical and Dental University, Tokyo, Japan; Institut de la Vision, France

## Abstract

In contrast to the uniform anatomy of the cerebellar cortex, molecular and physiological studies indicate that significant differences exist between cortical regions, suggesting that the spiking activity of Purkinje cells (PCs) in different regions could also show distinct characteristics. To investigate this possibility we obtained extracellular recordings from PCs in different zebrin bands in crus IIa and vermis lobules VIII and IX in anesthetized rats in order to compare PC firing characteristics between zebrin positive (Z+) and negative (Z−) bands. In addition, we analyzed recordings from PCs in the A2 and C1 zones of several lobules in the posterior lobe, which largely contain Z+ and Z− PCs, respectively. In both datasets significant differences in simple spike (SS) activity were observed between cortical regions. Specifically, Z− and C1 PCs had higher SS firing rates than Z+ and A2 PCs, respectively. The irregularity of SS firing (as assessed by measures of interspike interval distribution) was greater in Z+ bands in both absolute and relative terms. The results regarding systematic variations in complex spike (CS) activity were less consistent, suggesting that while real differences can exist, they may be sensitive to other factors than the cortical location of the PC. However, differences in the interactions between SSs and CSs, including the post-CS pause in SSs and post-pause modulation of SSs, were also consistently observed between bands. Similar, though less strong trends were observed in the zonal recordings. These systematic variations in spontaneous firing characteristics of PCs between zebrin bands in vivo, raises the possibility that fundamental differences in information encoding exist between cerebellar cortical regions.

## Introduction

The circuitry and the physiological properties of a brain region's cellular constituents constrain its computational properties. The cerebellar cortex offers what appears to be perhaps a unique opportunity for deriving structure-function correlations, because, with few exceptions, it is an essentially anatomically uniform structure whose few morphologically distinct cell types are interconnected according to relatively simple geometric rules. This uniformity is not absolute; for example, unipolar brush cells are limited primarily to so-called vestibulocerebellar regions [1]; nevertheless, the microcircuitry of most cerebellar cortical regions appears to be quite alike, suggesting that most cortical regions perform similar computations, and indeed, theories of cerebellar function have tended to be global in nature and based largely on a generic anatomical circuit diagram [2]–[7].

Belying this apparent uniformity, it has become increasingly clear that cerebellar cortical regions vary considerably on a genetic/molecular level [8]–[11]. A number of genes are expressed only in specific areas, and, indeed, the differential expression patterns of various molecules have been used to subdivide the cortex into discrete compartments [8], [11].

Among the various gene expression patterns that can be used to compartmentalize the cerebellar cortex that of zebrin II (aldolase C) is the most well-characterized. In mammals and birds, regions in which Purkinje cells (PCs, the sole output neurons of the cerebellar cortex) express zebrin (Z+) alternate with ones that do not (Z−), subdividing much of the cerebellar cortex into longitudinally running alternating Z+ and Z− bands [11]–[15]. This zebrin banding pattern is particularly useful as a frame of reference because it shows at least some correspondence with the topographies of the afferent (particularly the olivocerebellar zonal system) and efferent projections to and from the cortex [8], [16]–[24]. Moreover, the expression patterns of a number of other genes can be straightforwardly mapped onto that of zebrin, and many of these likely affect neuronal activity. For example, excitatory amino acid transporter 4 (EAAT4), which is preferentially localized to perisynaptic regions of the PC dendritic plasma membrane, is much more highly expressed in Z+ than Z− PCs [25], [26], and loss of its activity significantly enhances both parallel fiber and climbing fiber evoked EPSCs [27]–[32].

Such results suggest that the spiking patterns of PCs could vary significantly between cerebellar cortical regions, which, in turn, could lead to differences in the integrative/computational properties between local cortical regions, and potentially to differences in the functional states of the targets of these local cortical regions. Yet, the issues of whether the spontaneous spiking patterns of cerebellar cortical regions vary systematically between molecularly-defined compartments, and the behavioral consequences of such variation, remain unknown. Here we focus on the first part of this issue, and show that spontaneous PC activity varies systematically between Z+ and Z− bands of the cerebellar cortex.

Zones defined on the basis of physiological responses to stimulation of cerebellar afferents represent a second well-established scheme for compartmentalizing the cerebellar cortex [33]–[35]. Thus, we also looked for differences in PC firing patterns between such zones. A large degree of overlap between physiologically-defined zones and zebrin bands exists, and so, a priori, similar results should be found using both compartmentalization criteria. Yet, although the physiological zones and zebrin bands overlap extensively, they are not congruent, and thus comparisons between these two schemes could reveal fundamental organizational principles of cerebellar function.

In sum, a significant number of differences in the molecular and physiological properties of Z+ and Z− bands have been described. Such differences make it plausible that PC spiking activity should also vary between Z+ and Z− bands. Here, we report on the differences in spontaneous in vivo firing patterns of PCs between Z+ and Z− bands, and compare them with similar findings between different physiological zones.

## Methods

Experiments were performed in accordance with the NIH's *Guide for the Care and Use of Laboratory Animals* and the UK Animals (Scientific Procedures) Act 1986. Experimental protocols were approved by the Institutional Animal Care and Use Committees of New York University School of Medicine and the institutional Ethical Review Group at the University of Bristol.

### General surgical procedures

In most experiments in which the zebrin band of the recorded PCs was determined, female Sprague-Dawley rats (225–300 g) were initially anesthetized with ketamine (100 mg/kg) and xylazine (8 mg/kg) intraperitoneally. Supplemental anesthetic was given via a femoral catheter (ketamine, ∼260 µg/kg/min; xylazine, ∼50 µg/kg/min) to maintain a constant depth of anesthesia. In the other experiments urethane was used as the anesthetic and an initial dose of 1.2–1.5 g/kg (i.p.) was given with supplemental doses given if needed. In experiments where PCs were assigned to physiologically-identified cerebellar cortical zones, recordings were obtained from male Wistar rats anesthetized with ketamine (100 mg/kg) and xylazine (5 mg/kg) intraperitoneally, and supplementary doses of anesthetic were administered as required. In all experiments, the depth of anesthesia was regularly assessed by a paw pinch to monitor reflex muscle tone. Rectal temperature was maintained at 37°C. To gain access to the cerebellum, animals were placed in a stereotaxic frame, and a craniotomy performed to expose the posterior lobe of the cerebellum.

### Recording and localization of PCs

#### Zebrin band experiments

Following removal of the dura, the cortical surface was stabilized and protected by covering it with an electron microscope grid pre-embedded in a thin sheet of silicone rubber. The grid was cemented onto the skull surrounding the craniotomy. Extracellular recordings of PC activity at the somatic level were obtained from the apices of crus II and vermis lobule VIII using glass microelectrodes filled with 2.0 M NaCl solution and mounted on a motorized 3D manipulator (MCL-3, Lang GmbH & Co. KG). A 3 to 5-min continuous recording of spontaneous activity was made when simple spikes (SSs) and complex spikes (CSs) were both observed, usually at a depth between 200 and 300 µm. The presence of SSs and the initial positivity of the CS waveform indicated that the recordings were made at or close to the PC soma. Neural activity was recorded using a multichannel recording system (MultiChannel Systems, Germany) with a 25 kHz/channel sampling rate, gain of 1000x, and band pass filters set at 0.2–8.0 kHz.

Recordings of dendritic CSs from a dataset that was used for other purposes in a previous study were also analyzed [36]. In these experiments a multielectrode recording approach was used in which CS activity was obtained from arrays of crus 2a PCs simultaneously. The surgical procedures were essentially identical to those just described for the somatic recordings. The electrodes used in the multielectrode experiments were filled with a 1∶1 mixture of 2.0 M NaCl and glycerin (the addition of glycerin slowed evaporation of the internal electrode solution). Details of the construction and implantation of the electrodes can be found in [37]. In brief, individual electrodes were generally implanted to depths of 100–150 µm below the brain surface, corresponding to mid-molecular layer levels, and then were released from the micromanipulator. Once all electrodes in the array have been implanted CSs were recorded simultaneously from all electrodes for a 20-min period. It should be noted that SSs are not observed when recording CS activity from the middle molecular layer. This is consistent with the poor back propagation of somatic sodium spikes into the dendrites [38].

**Figure 1:**
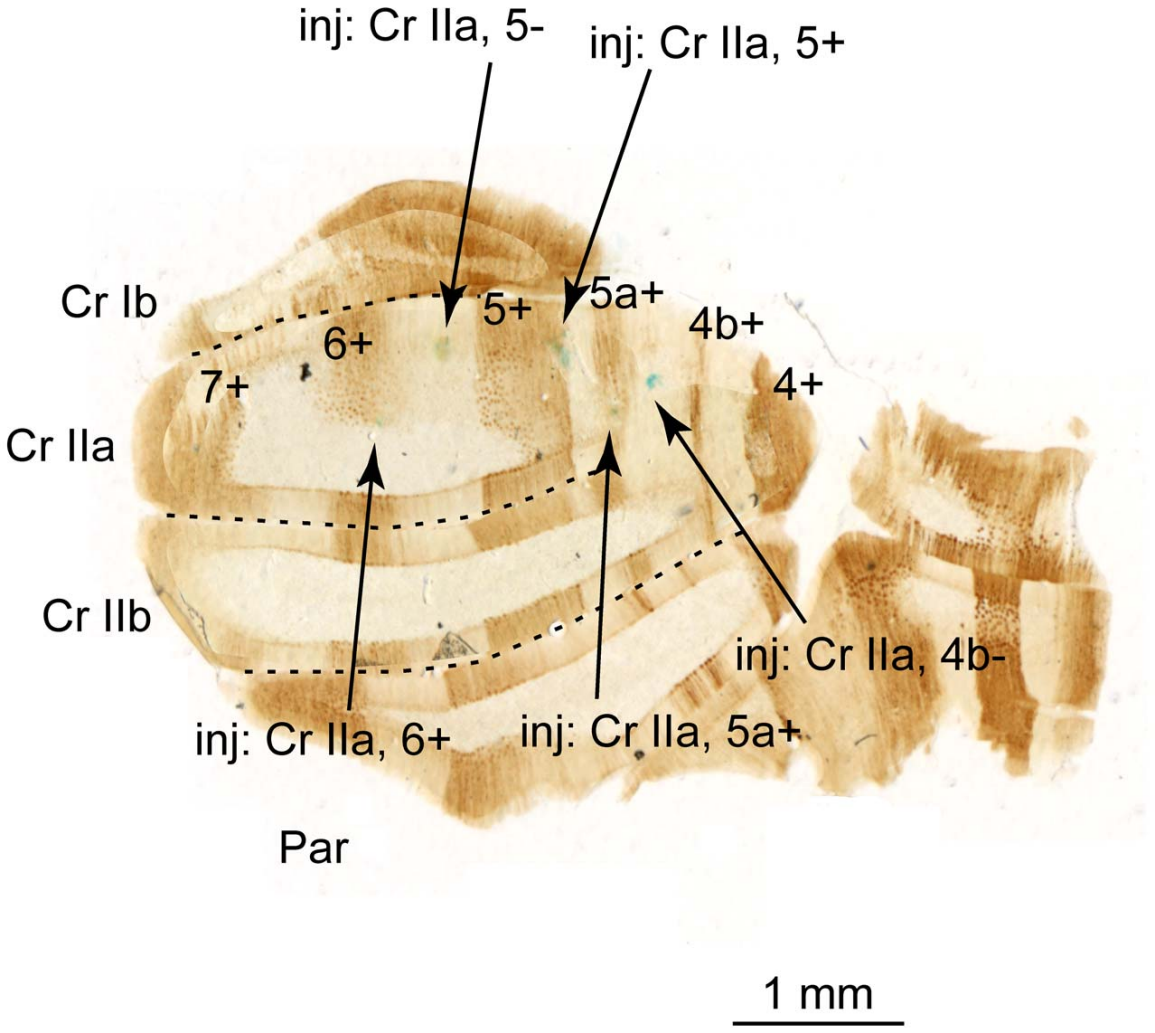
Zebrin staining of the hemisphere of the rat cerebellum viewed in coronal section. Sulci between lobules indicated by dashed lines. The alcian blue dye spots used to locate the PC recording sites are marked with arrows (location of spot indicated by label at tail end of arrow). Z bands 4+ to 7+ are labeled. Abbreviations: Cr =  crus; Par, paramedian; and inj  =  injection site.

At the end of every experiment, the recording pipette was drained of the NaCl solution, refilled with alcian blue solution, and connected to a pressure-injection system, while the pipette remained in place on the microdrive. Injections were then made either at the corners of the recording platform, or, when only a few PCs were recorded in the experiment, to the location of each recorded PC based on its coordinates relative to an origin point defined on the surface of the lobule. To make the injection, the pipette was lowered into the cortex to a depth of 200–300 µm and pressure applied to eject a small amount of the dye solution. The animal was then intracardially perfused with phosphate buffered saline (PBS) followed by 4% paraformaldehyde. The brains were embedded in gelatin and cut in the plane parallel to the cerebellar surface of the recording area into 80 µm-thick serial sections. This thickness (unusually large for immunostaining) was used to locate the injection marks in a small number of sections. All serial sections were then processed for zebrin staining as previously described [12], [13]. Briefly, sections were incubated with the biotinylated rabbit anti-Aldoc antibody (#69076, immunogen: amino acids 322–344 of rat Aldoc; produced in one of the laboratories and tested for specificity with Western blot [12]; 320 ng/ml) for 48 hours, then with biotinylated peroxidase-avidin complex (PK6100 Elite ABC kit; Vector Laboratories, Burlingame, CA) for 8–12 hr, and finally incubated with diaminobenzidine (0.5 mg/ml), glucose oxidase (0.01 mg/ml; type II, G-6125; Sigma, St. Louis, MO), ammonium chloride (4 mg/ml), and beta-D(+)-glucose (2 mg/ml) in phosphate-buffered saline for 60 min. Sections were mounted on glass slides, dried and coverslipped with Permount (Fisher Scientific, Fair Lawn, NJ). Serial sections were photographed with 4x objective. Image files of photos were trimmed at the cerebellar surface and superimposed by referring to the folial contour and the labeled zebrin bands by using graphics software (Illustrator, Adobe, San Jose, CA, U.S.A.). The location of the recording sites relative to zebrin bands in crus II or lobule VIII was determined based on the stained zebrin bands and recovered dye marks, which emerged in the superimposed assembly of photographs, and the coordinates of the recording sites (Fig. 1). Due to uneven shrinkage of the tissue, and the narrowness of some zebrin bands, not all cells could be confidently localized to a specific zebrin band. Only cells located with high confidence were included in the analyses.

#### Zonal experiments

Recordings of PCs in this study were from a dataset that was used previously [39]. Glass-insulated tungsten microelectrodes (Alpha-Omega, Israel) were used to record PC activity extracellularly. The recordings of individual PCs lasted approximately 30 min on average. Waveforms were filtered between 0.3–5.0 kHz. Recordings were digitized on-line (sampling rate, 21 kHz) using a Cambridge Electronic Design (CED, Cambridge, UK) 1401 analog-to-digital converter and Spike2 software (CED).

Climbing fiber fields were first evoked by peripheral stimulation and recorded using a surface electrode in order to map the approximate positions of the physiologically-identified A2 and C1 zones as a guide for the single unit recordings (for details see [40]). In brief, bipolar percutaneous stimulating electrodes were inserted into the contralateral whisker pad and the ipsilateral forelimb. Stimuli were given (single pulse; 0.1 ms duration, 1 Hz) at an intensity sufficient to evoke a small but visible muscle twitch from the stimulated body part.

The criteria used to classify individual PCs as located within a particular cerebellar cortical zone were as follows: (i) If the recording track was located no more than 0.6 mm lateral from the paravermal vein, (ii) less than 2.5 mm from the cortical surface, and (iii) the cell responded to contralateral face stimulation with an increase in CSs with an onset latency <30 ms, then it was classified as located in the A2 zone. Similarly, if the recording track was (i) between 0.6–1.3 mm lateral from the paravermal vein, (ii) less than 2.5 mm from the cortical surface, and (iii) the cell responded to ipsilateral forelimb stimulation with an increase in CSs with an onset latency <25 ms, then the PC was classified as located in the neighboring C1 zone.

### Data Analysis

In zebrin band experiments, spikes were detected and sorted offline using Igor Pro (Wavemetrics, Lake Oswego). For PCs recorded in the zonal experiments, SS and CS activity was discriminated independently via a template matching algorithm (Principal component analysis, Spike2). In all experiments cross-correlation between the firing time of SSs and CSs was performed to ensure that they were recorded from the same PC, based on the presence of a post-CS pause in SS firing. The pause was measured as the latency from the onset of a CS to the first SS that occurred following that CS. Subsequent analyses of zebrin band experiments were performed using custom routines in Igor Pro, Matlab (Mathworks, Natick), and Excel (Microsoft). Analysis of zonal experiments was performed using Neuroexplorer (Nex Technologies, Madison, AL) and Prism (Graphpad, La Jolla).

Some of the data were found to be normally distributed, but others were not. Thus, for the sake of simplicity and consistency, a non-parametric Mann–Whitney U-test was used for testing the statistical significance and medians are presented. In rare cases, which are noted in the text, when the data were normally distributed and the parametric t-test gave a different result than the non-parametric test, the results of the t-test (and mean ±s.d.) are also presented and took precedence.

The duration of the post-CS pause in SSs is different in Z+ and Z- bands (see Results). To investigate how much of this difference was due to differences in baseline SS firing rates between bands we first calculated the expected pause duration as if it were simply a function of SS firing rate; that is, under the assumption that CSs and SSs occur independently. Deviations from this value could then be used as a measure of the active suppression that was present.

The expected pause value under the assumption of independence equals one half the mean SS interspike interval (ISI_SS_), which can be shown as follows. Take two successive SSs (SS_k_ and SS_k+1_) separated by the time interval, t_i_, with a CS occurring between them. Since the CSs and SSs are independent, the CS occurs with equal probability at any point in the interval t_i_. Thus, the probability of the CS occurring at time t is Pr(t)  = 1/t_i_ and the average time of the CS in the interval is given by
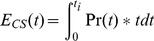
which evaluates to t_i_/2. The expected pause is then the difference from the average time of the CS to the second SS, t_i_–t_i_/2 = t_i_/2. In general, the ISI_SS_ will vary between SSs so that the average pause for a SS train will be given by:



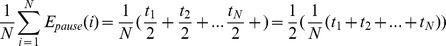



This sum, however, is simply half the mean of all ISI_SS_ that contain a CS. Given the assumption of independence these ISIs should represent a random sample of all ISI_SS_, and thus be an unbiased estimate of the mean ISI_SS_ of the complete SS population. The ratio of the SS pause to the mean ISI_SS_ is therefore 0.5.

## Results

### Database

This study presents results related to differences in PC activity between Z+ and Z− bands using four specific datasets, the first three consist of recordings of PC activity under ketamine/xylazine anesthesia. The first set consists of crus IIa and vermis lobule VIII PCs whose locations were verified histologically using reconstructed zebrin maps (n = 38 PCs, 11 animals) (see Methods, ‘Recording and localization of PCs’). A second dataset of recordings of SS and CS activity were analyzed from PCs whose locations were physiologically identified to be in the A2 or C1 zones (n = 26 PCs, 14 animals). Although the physiological zones and zebrin bands are not entirely congruent in the posterior lobe of the rat cerebellum, the A2 zone comprises mainly Z+ bands whereas the C1 zone comprises mainly Z− ones [17]. Because these zones are not fully equivalent to the zebrin bands, we present the data from the zebrin and zonally-identified PCs separately for each analysis. Furthermore, later we present anatomical and statistically analyses in support of the validity of using the A2 and C1 zones as surrogates for Z+ and Z− bands (see later Results subsection ‘*Degree of overlap of physiologically-defined zones with zebrin bands’*). The third dataset consists of multielectrode recordings from a prior study [36], which, because of electrode placement in the mid to upper molecular layer (see Methods), consists only of CS activity. In this dataset PC locations with respect to the zebrin bands were verified histologically as described in the Methods. The fourth dataset consists of PC activity from lobules VIII and IX that was recorded and identified in exactly the same manner as described for the first dataset, except that urethane was used as the anesthetic.

**Figure 2:**
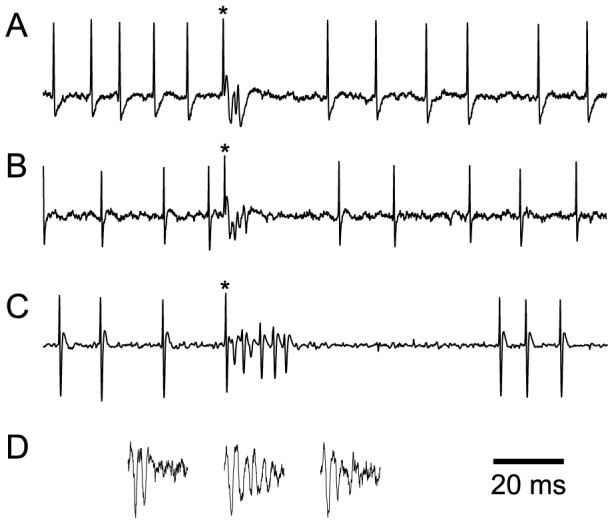
Extracellular recordings of PC activity. (A–B) Sample recordings from zebrin localized PCs recorded under ketamine/xylazine (A) and urethane (B) that show both SSs and a CS (indicated by ‘*’). Note the pause in SSs following the CS. (C) Recording of PC from the zonal database displaying both SSs and a CS (indicated by ‘*’). Again note the pause in SSs following the CSs. The recording in C was filtered with a higher cutoff frequency for the high pass filter, eliminating the slower potential that can be seen in the recordings in (A and B), and on which the spikelets ride. (D) Extracellular traces showing three CSs from recorded from a PC with the electrode in the molecular layer, approximately 100 µm below the folial surface of crus 2a. Note how the number and size of the spikelets varies between CSs, but that the size and shape of the initial spike are nevertheless relatively constant, indicating that a single unit is being recorded.

The specific locations of all recordings are listed in Table 1. Examples of the recordings in each dataset are shown in figure 2. In the presentation below we describe the results from the ketamine/xylazine recordings (data sets 1, 2 and 3). In the final Results subsection we compare these results with those from the urethane recordings.

**Table 1:** Spatial distribution of the recorded cells.

A.	Zebrin bands	Somatic	Dendritic	All
	Ketamine/Xylazine			
	Crus IIa 4−	1	3	4
	Crus IIa 4b−	2	4	6
	Crus IIa 4b+	1		1
	Crus IIa 5−	10	13	23
	Crus IIa 5+	5	20	25
	Crus IIa 5a−	2		2
	Crus IIa 5a+	3		3
	Crus IIa 6−		8	8
	Crus IIa 6+	4	15	19
	Crus IIa 7+		2	2
	Lobule VIII 1−	2		2
	Lobule VIII 1+	1		1
	Lobule VIII 2+	3		3
	Urethane			
	Lobule VIII 1−	2		2
	Lobule VIII 2+	2		2
	Lobule VIII 2−	1		1
	Lobule VIII 3+	1		1
	Lobule VIII 3−	1		1
	Lobule VIII 4−	1		1
	Lobule IX 2+	11		11
	Lobule IX 3+	4		4
	Sum	57	65	122

B.	Physiological zones	Somatic
	Crus II A2	13
	Crus II C1	1
	Paramedian A2	5
	Paramedian C1	4
	Copula Pyramidis C1	3
	Sum	26

A) The zebrin bands for all PCs were histologically identified. Some of the cells were somatically recorded; others dendritically. B) The zonal locations of an additional sample of PCs were physiologically determined. All cells were somatically recorded. A2 zone comprises mainly Z+ bands while the C1 zone comprises mainly Z− bands. Two cells recorded in Crus II A2 zone did not fire SSs and were not included in the analysis of SS firing rate.

### PCs in Z− bands show higher SS firing rates

**Figure 3:**
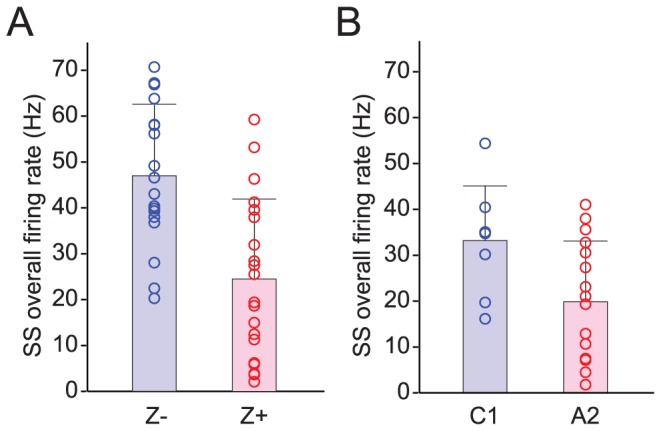
Z− PCs have higher SS firing rates. The difference in SS firing rate is consistent for both histologically (A) and physiologically (B) located PCs. Circles represent the overall firing rates of individual PCs in Z− bands or C1 zone (blue) and in Z+ bands or A2 zone (red). In each distribution, the bar indicates the mean. Error bars indicate one SD of the distribution.

Comparison of SS average firing rates (total number of SSs divided by recording duration) between PCs in Z+ and Z− bands revealed a significant difference between these populations. Under ketamine, Z− cells fire at rates that are 92% higher, on average, than Z+ cells (Z− = 44.8 Hz, n = 18; Z+ = 22.5 Hz, n = 20; p = 0.0006). Figure 3A plots the entire distribution of SS firing rates of somatically-recorded, zebrin-identified PCs, and shows that while the distributions of the two PC populations overlap, the Z− distribution is shifted toward higher firing rates.

We also examined PCs recorded in the A2 and C1 zones in medial aspects of crus II, paramedian lobule, and copula pyramidis, and found a similar difference in the average SS firing rates of PCs between the two zones. SS rates for C1 (mainly Z−) were significantly higher (67%) than for A2 (mainly Z+), consistent with our first dataset (Fig. 3B; medians: C1 = 34.9 Hz, n = 8; A2 = 20.2 Hz, n = 16; p = 0.0537, Mann–Whitney U-test; means: C1 = 33.2±11.9 Hz, n = 8; A2 = 19.9±13.2 Hz, n = 16; p = 0.022, t-test; note that in two cells from the A2 zone only CSs were recorded and so these PCs were not included in this analysis).

If the two datasets are combined, the difference between the Z+/A2 and Z−/C1 groups remains highly statistically significant (Z−/C1: 40.1 Hz, n = 26; Z+/A2: 20.2 Hz, n = 36; p∼0). In sum, the results as a whole indicate that SS rates are higher in Z− bands than in Z+ ones throughout much, if not all, of the posterior lobe.

### The regularity of SS activity varies between Z+ and Z− bands

Regularity of SS activity may be a critical parameter governing the efficacy of transmission of information to the cerebellar nuclei, particularly if PCs use a rate code, as is often assumed (e.g., see [5]). Thus, we investigated whether the regularity of SS activity varied between Z+ and Z− bands, as any differences might point to differences in the way Z+ and Z− cortical bands interact with their target cerebellar nuclear regions.

**Figure 4:**
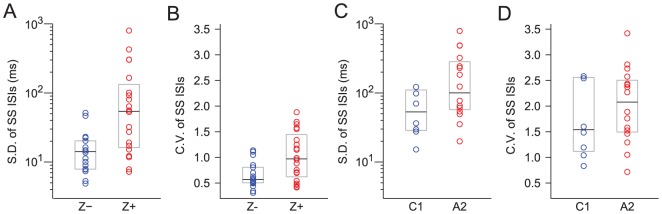
Z− PCs have more regular SS trains than do Z− PCs. Plots of the SD (A) and CV (B) of the ISI distributions for SS trains from Z+ and Z− PCs. Analogous plots for the zonal data are shown in (C) and (D). In each plot data the circles represent values from individual PCs, the horizontal lines show the medians of the various distributions, and the boxes indicate the interquartile ranges.

We measured the absolute and relative regularity of the SS trains using the standard deviation and coefficient of variation (CV = SD/mean), respectively, of the interspike interval (ISI) distribution. The spike trains of Z+ PCs were more irregular in both absolute and relative terms (Fig. 4A, B; SDs: Z− = 14.2 ms, n = 18; Z+ = 54.3 ms, n = 20; p = 0.0018; CVs: Z− = 0.57, n = 18; Z+ = 0.97, n = 20; p = 0.0172).

For the zonal data, trends consistent with the zebrin data were found, although these differences were not statistically significant (Fig. 4C, D; SDs: C1 = 53.6 ms, n = 8; A2, 101.0 ms, n = 16; p = 0.0708; CVs: mainly Z− C1 = 1.54, n = 8, mainly Z+A2 = 2.08, n = 16, p = 0.4812).

### CS firing rate relationship to Z bands

**Figure 5:**
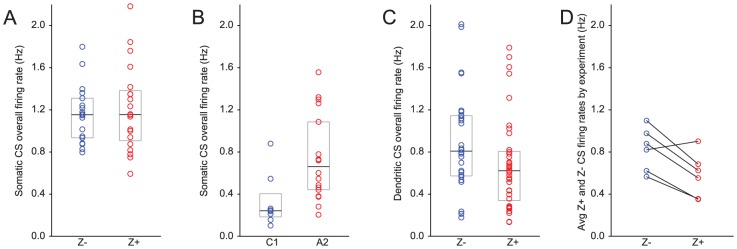
Variable relationship of CS firing rate to Z+ and Z− bands. (A–C) Distribution of CS firing rates for Z− (or C1) and Z+ (or A2) cells. Circles represent the firing rates of individual PCs. In each distribution, the box indicates the 1st and 3rd quartile and the black line represents the median of the distribution. (A) Somatically recorded Z+/− PCs show comparable CS firing rate. (B) Somatically recorded PCs in C1 zone show significantly lower firing rates than those in A2 zones. (C) Dendritically recorded PCs in Z− bands had a higher median rate than Z+ bands despite the wide scatter of values within each type of band. (D) Median firing rates compared between Z+ and Z− bands within individual experiments. Each circle represents median CS firing rate for all Z− (blue) or Z+ (red) PCs from an individual experiment. Lines connect the Z+ and Z− cells obtained in the same experiment.

It is known that CS and SS rates can influence each other, leading to either an inverse or a direct correlation in rates, depending on the experimental manipulation [41]–[47]. Thus, given the observed differences in SS firing rates between Z+ and Z− bands we next investigated whether CS firing rates also varied. In contrast to the SS results, those for CSs were not consistent between datasets. For the somatic recordings from PCs in zebrin-identified locations, the distribution of firing rates overlapped substantially with no statistical difference in the medians (Fig. 5A; Z− = 1.15 Hz; n = 18; Z+ = 1.16 Hz, n = 20; p = 0.92). In contrast, in recordings from zonally-identified PCs, A2 cells showed significantly higher CS firing rates than did C1 cells (Fig. 5B; C1 = 0.24 Hz, n = 8; A2 = 0.66 Hz, n = 18; p = 0.0083).

It is perhaps not surprising that the results from these two datasets differ with regard to CS firing rates, as CSs are generated in response to activity arising in inferior olivary neurons rather than by local cerebellar cortical network activity, and thus CS firing rates may be less completely determined by factors related to the molecular make up of zebrin bands than are SS rates. In particular, CS firing rates not only are influenced by GABAergic inhibitory inputs from the cerebellar nuclei (whose activity is indeed modulated by PC activity), but also by non-GABAergic, presumably glutamatergic inputs from extra-cerebellar regions [48].

Thus, to determine the relationship between CS firing rates and zebrin, if any, it would be ideal to hold these other potential variables constant. While we could not do that, we examined CS activity recorded from PCs that were recorded simultaneously, and hence under an identical brain state (dataset 3) [36]. The CS firing rates in this larger dataset overlapped substantially with those of the somatically recorded CSs, with median CS firing rates that fell in between those of the two somatic datasets (Fig. 5C; Z+ = 0.62 Hz, n = 37; Z− = 0.81 Hz, n = 28). A small but statistically significant difference in CS average firing rates between Z+ and Z− PCs was found (p = 0.0499); however, in this case it was the Z− PCs that showed the higher rates. Although the difference was small, it was generally consistent across experiments, as shown by comparing the median CS firing rates for pairs of Z+ and Z− PCs obtained in the same experiment (Fig. 5D). In five of six experiments rates were higher for Z− cells (p = 0.018, paired t-test).

In sum, there were mixed findings relating to CS firing rates in relation to zebrin bands. However, CSs also have a profound influence on subsequent SS activity and the following sections consider this aspect of CS activity in more detail.

### Differences in the post-CS pauses in SS activity between Z+ and Z− bands

One of the distinctive interactions between SSs and CSs is the pause in SS activity following most CSs [49]–[51]. The post-CS pause has also been suggested to be a potentially important mechanism for distinguishing CS and SS activity by the cerebellar nuclei [52]. Thus, we investigated whether the characteristics of the pause varied between Z+ and Z− bands.

**Figure 6:**
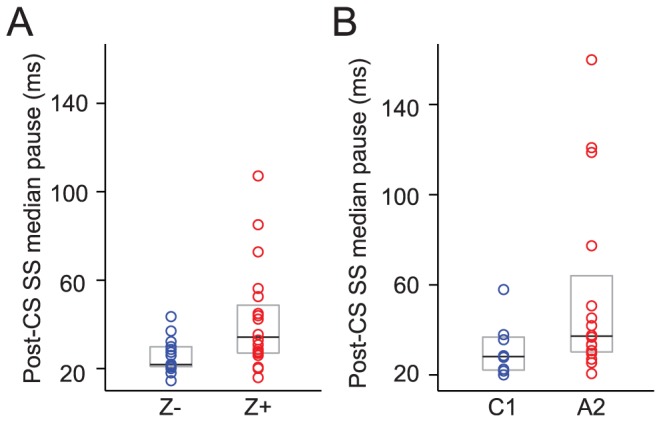
PCs in Z+ bands show longer absolute post-CS pauses in SS than those in Z− bands. The difference is consistent for both histologically (A) and physiologically (B) located PCs. Each circle represents the median duration of the pause of individual PCs in Z− bands or C1 zone (blue) and in Z+ bands or A2 zone (red). The black lines indicate the overall median across each group, and the grey boxes indicate the range from 25% to 75% of each distribution. Note that Z+ bands and A2 zone also consistently show higher variation than Z− bands and C1 zone.

The interval between each CS and the SS immediately following it was measured and the median pause duration was calculated for each cell. The population median pause duration of PCs in Z+ bands were found to be 56.5% longer than those in Z− bands (Z− = 21.9 ms, n = 18; Z+ = 34.2 ms, n = 20; p = 0.0068; Fig. 6A). In addition, as a population, PCs from Z+ bands showed a much wider distribution in the cell-to-cell variation of pause duration than did PCs from Z− bands, even when this variation was normalized to account for the differences in the mean pause duration of the Z+ and Z− populations (CV: Z+ = 0.554; Z− = 0.288).

Analysis of the zonal dataset showed results consistent with the zebrin data. A2 PCs (mainly Z+) showed longer pauses than did C1 PCs (mainly Z−) (C1 = 28.2 ms, n = 8; A2 = 37.2 ms, n = 16); however, the difference in medians did not reach statistical significance (p = 0.0922, Mann–Whitney U-test). Nevertheless, as shown in Fig. 6B, the difference in the distribution of pause durations between A2 and C1 cells shows good agreement with the Z+ and Z− data, respectively. Also consistent with the zebrin data, was the finding that the normalized variation in pause duration among A2 PCs was higher than that among C1 PCs (CV: A2, 0.747; C1, 0.396).

For the zebrin data, we next analyzed factors that could contribute to the significant difference in pause duration. First we investigated the effect of the slower SS firing rates of Z+ PCs. With a slower average firing rate the duration of the time between a CS and the immediately succeeding SS will be greater on average. Specifically, assuming that the occurrences of SSs and CSs are independent, the expected latency of a SS following a CS is equal to half the mean SS ISI (see Methods). Secondly, we investigated whether any of the difference in post-CS pause duration between Z+ and Z− bands involved differences in active suppression of SS activity.

**Figure 7:**
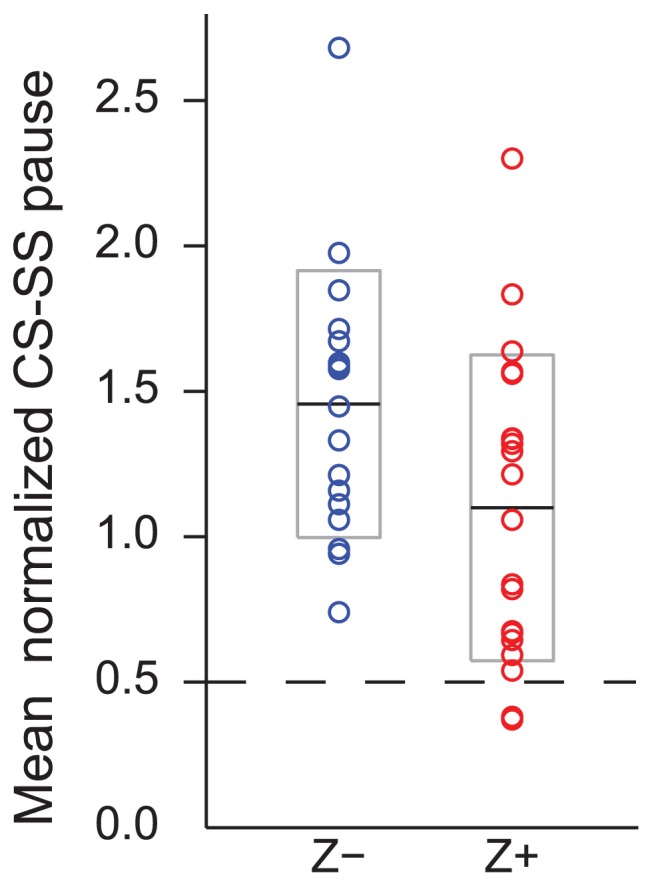
CSs in Z− bands cause a relatively stronger active suppression of SSs. The mean SS pause duration of each PC was normalized by dividing by its mean SS ISI. The distribution of ratio is shown for Z+ and Z− PCs. Dashed line indicates the expected ratio if the latency from a CS to the first succeeding SS is solely a function of SS firing rate. The solid black lines indicate the overall median across each group, and the grey boxes indicate the range from 25% to 75% of each distribution.

To separate the contribution of these two factors to the difference in pause duration between Z+ and Z− bands, we normalized the mean SS pause duration of each PC by the mean SS ISI. This ratio should equal 0.5 (pause  = ISI/2, so pause/ISI = 1/2) if the entire pause in SSs is simply a function of SS firing rate. The majority of PCs in both Z+ and Z− bands show ratios that are higher than 0.5 (Fig. 7), which indicates that the pause in SSs is longer than would be expected if the pause duration is based solely on SS firing rate. Thus, there is active suppression of SSs following a CS in both Z+ and Z− bands. However, the ratios of the Z− PCs are significantly higher than those of the Z+PCs (Z− = 1.51, n = 18; Z+ = 1.14, n = 20; p = 0.0317), indicating a greater active suppression of SSs by CSs in Z− bands.

In sum, although the absolute pause duration is greater in Z+ PCs, this is largely attributable to the lower SS firing rates (i.e., longer mean ISIs) in those PCs, because when mean ISI is controlled for, Z− PCs show a greater relative pause in SS activity following a CS.

### PCs in Z+ bands have a stronger increase in SS local activity after CS

**Figure 8:**
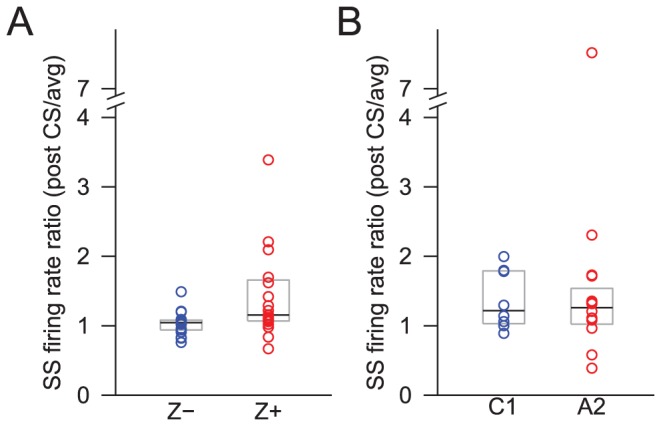
Stronger increase in SS activity after CS in Z+ than Z− bands. The difference is consistent for both histologically (A) and physiologically (B) located PCs. Each circle represents the median ratio of the SS firing rate for the 100-ms period after a CS to the overall SS firing rate for the individual PCs in the Z− bands or the C1 zone (blue) and in the Z+ bands or the A2 zone (red). The black lines indicate the overall median across each group, and the grey boxes indicate the range from 25% to 75% of each distribution.

After the initial pause in SSs following a CS, there is often a longer latency modulation in SSs, which may take the form of a delayed return to baseline activity or a transient overshoot in activity [53]. To test whether the post-pause modulation differs between Z+ and Z− PCs, we measured the average firing rate of SS in a 100-ms time window that started at the onset of each CS. This post-CS firing rate was normalized by the overall SS firing rate of the same cell to provide a ratio that reflected the post-CS change in SS firing rate relative to the overall spiking level of the cell. This ratio was significantly different between Z+ and Z- cells (Fig. 8A; Z− = 1.05, n = 18; Z+ = 1.16, n = 20; p = 0.0065).

Thus, in Z+ bands there is a stronger positive modulation of SS activity following each CS; this relative increase in activity occurred despite the longer initial pause of PCs in Z+ bands. Furthermore, the CV of the distribution of ratio values was much wider for Z+ cells than for Z− cells (Z− = 0.160, Z+ = 0.437; or 1∶2.7), indicating that PCs in Z+ bands are more heterogeneous with regard to how SSs and CSs interact.

PCs in the A2 and C1 zones showed a similar differential in their post-pause modulation of SSs to the Z+ and Z− PCs (Fig. 8B). That is, A2 PCs had a relatively higher modulation of SS levels than did C1 PCs, but the difference in the modulation ratios was not statistically significant (C1 = 1.22, n = 8; A2 = 1.26, n = 16; p = 0.8303). The CV of the ratio value for the A2 cell population was larger than that for the C1 population (C1 = 0.31, A2 = 1.04; or 1∶3.4), consistent with the zebrin data.

### Differences in Z+ and Z− PC activity under urethane

To rule out the possibility that the observed differences between Z+ and Z− PCs reflect the particular anesthetic state in which the recordings were obtained we investigated whether similar differences between Z+ and Z− PCs existed under urethane, an anesthetic that acts via different mechanisms than ketamine/xylazine. Recordings were obtained of Z+ and Z− PCs from vermis lobules VIII and IX (Z−, n = 5; Z+, n = 18; for details on lobular and zebrin band distribution see Table 1). With one exception, all of the parameters that showed significant differences between Z+ and Z− under ketamine/xylazine showed identically-directed differences under urethane. Specifically, under urethane, statistically significant differences were found for SS firing rate (Z− = 44.2 Hz; Z+ = 21.0 Hz; p = 0.0009), the SD of the SS ISI (Z− = 6.67 ms; Z+ = 14.47 ms; p = 0.0041), and the post-CS pause in SSs (Z− = 40.9 ms; Z+ = 82.4 ms; p = 0.0279). For the CV of the SS ISI distribution, the difference between Z+ and Z− cells was not significant (Z− = 0.272; Z+ = 0.370; p = 0.1679). The post-pause modulation of SS activity in the 100 ms post-CS period could not be compared with the values under ketamine/xylazine because the pause duration under urethane was longer than half of this period in most PCs (hence no spikes were present for at least the first half of the period). The differences in the various parameters between Z+ and Z− PCs are summarized in the top halves of Tables 2A and B (labeled ‘Global’), and show the consistency of the results the three data sets where SS activity was recorded. That is, for each parameter analyzed (except CS firing rate), the inequality sign points in the same direction for all three datasets (Table 2B, Global).

**Table 2:** Comparison of differences between Z− and Z+ regions by region and anesthetic.

(A)								
	N	SS FR	SD	CV	CS FR	Pause	NPause	PPause
		(Hz)	(ms)		(Hz)	(ms)		
Global								
Zebrin K/X	18/20	44.8/22.5	14.2/54.3	0.57/0.97	1.15/1.16	21.9/34.2	1.51/1.14	1.05/1.16
Zebrin U	5/18	44.2/21.0	6.7/14.5	0.27/0.37	0.36/0.88	40.9/82.4	1.96/1.47	n/a
Zonal K/X	8/16^a^	34.9/20.2	53.6/101.0	1.54/2.08	0.24/0.66	28.2/37.2	2.47/1.38	1.22/1.26
Regional								
cIIA K/X	15/13	49.2/32.0	13.7/19.1	0.56/0.71	1.13/1.14	21.7/27.7	1.59/1.29	1.04/1.15
VIII K/X	3/7	28.1/5.9	22.4/165.1	0.63/1.34	1.22/1.35	32.1/56.2	1.16/0.67	1.08/1.62
VIII U	5/3	44.2/29.8	6.7/12.8	0.27/0.41	0.36/0.77	40.9/83.0	1.96/2.63	n/a
PM K/X	5/4	37.4/10.7	32.2/231.8	1.34/2.57	0.2/0.72	97.3/159.8	2.47/1.38	1.10/1.32

(B)								
	N	SS FR	SD	CV	CS FR	Pause	NPause	PPause
Global								
Zebrin K/X	18/20	**>	**<	*<	≈	**<	*>	**<
Zebrin U	5/18	**>	**<	<	*<	*<	>	n/a
Zonal K/X	8/16^a^	*^b^>	^T^<	<	**<	^T^<	>	<
Regional								
cIIa K/X	15/13	*>	^T^<	<	≈	<	*>	*<
VIII K/X	3/7	*>	*<	*<	<	*<	>	<
VIII U	5/3	*>	*<	<	<	*<	<	n/a
PM K/X	5/4	^T^>	^T^<	<	<	<	>	<

(A) Summary of values for each parameter that was analyzed. The format is Z−/Z+. Global section gives the medians for the entire populations for the three data sets where somatic recordings were obtained. The Regional section provides data for each of the lobules where enough Z+ and Z− cells were recorded to make a comparison. K/X: ketamine/xylazine, U: urethane. cIIa: crus IIA, VIII: vermis lobule VIII, PM: paramedian lobule. SS FR = SS average firing rate; SD, CV = SD and CV of the SS ISI distribution; Pause: the median post CS-pause in SS activity; NPause =  the normalized pause in SS activity (mean Pause/mean SS ISI); PPause  =  increase in SS firing rate within 100 ms post-PC; a: 16 A2 cells included for most of the measurements except CS FR, for which 18 A2 cells were included, because 2 PCs in the A2 zone had no SS activity; (B) Summary of the direction and significance of the difference in parameter values between Z− and Z+ bands. The > and < symbols indicate cells in Z− regions have a larger or smaller value for each parameter, respectively. **: P<0.01; *: 0.01<P<0.05; T: 0.05<P<0.1; b: based on t-test instead of non-parametric test, see Methods.

### Zebrin-related differences are present in multiple regions

The above results suggest that the differences between Z+ and Z− PC firing patterns are a general phenomena, at least throughout much of the posterior lobe of the cerebellar cortex. To test the generality of the results further, we analyzed the differences between Z+ and Z− bands for individual lobules. For the zebrin experiments enough Z+ and Z− PCs were present in crus IIa and vermis lobule VIII to make comparisons. For the zonal recordings this was the case for the paramedian lobule.

In almost all cases, each individual lobule showed the same trends as were observed in the combined population (Table 2A,B, bottom halves labeled ‘Regional’). That is, relative to their Z+ counterpart, each Z− region had higher SS firing rates, greater absolute and relative regularity, shorter absolute but longer relative pauses, and a less positive post-CS modulation of SSs following the initial pause. In most cases these differences were statistically significant (indicated by * or ** in Table 2B). There was just one exception other than CS firing rate: the normalized pause duration in vermis VIII under urethane. Thus, the general consistency in the results across lobules and anesthetics suggests that the observed differences between PC activity in Z+ and Z− bands is a common organizational principle of the cerebellar posterior lobe, and possibly the entire cerebellum.

### Degree of overlap of physiologically-defined zones with zebrin bands

**Figure 9:**
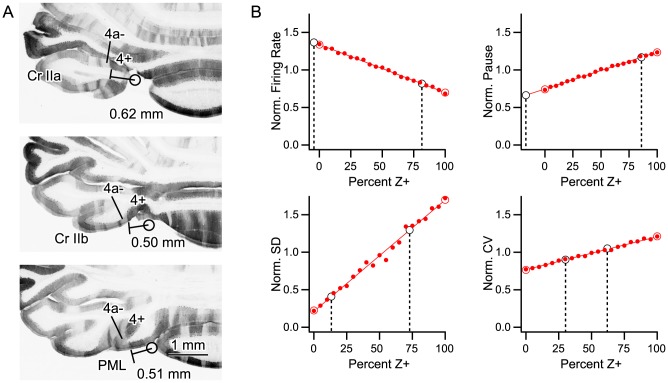
Zebrin composition of A2 and C1 zones. (A) Anatomical estimate of zebrin bands for A2 and C1 zones in crus II and paramedian lobule. In horizontal sections on which the apical area of crus IIa, crus IIb, and paramedian lobules were seen, the distance between the putative position of the paravermal vein and the boundary between Z+ band 4+ and Z− band 4a− was measured. Circle indicates the putative position of the paravermal vein. This boundary roughly corresponded to the physiological boundary between A2 and C1 zones (0.6 mm, Methods). (B) Plots of normalized spike train parameters (firing rate, SS pause, SD of ISI, and CV of ISI) for populations consisting of different percentages of Z+ PCs. Simulated population averages indicated by filled small red circles. Values for experimental zebrin datasets indicated by large unfilled red circles (Z− at 0%, Z+ at 100%). Lines are least squares fits to the simulated population data. Large unfilled black circles show zonal data (C1 shown by left circle in each plot, A2 by right circle). Placement of black circles along x-axis was determined using the experimental parameter value in the corresponding regression equation, and thus provided an estimate of the percentage of Z+ PCs in the population (indicated by dashed black lines).

To validate the use of the A2 and C1 zones, respectively, as surrogates for Z+ and Z− bands, we estimated the zebrin composition of these zones for the regions of cortex where PC activity was recorded. In zebrin (aldolase C) immunostained brains, Z+ band 4+ is generally located under the paravermal vein and extends up to 0.5–0.6 mm laterally in crus IIa, crus IIb and paramedian lobule (Fig. 9A). Since fixed tissue for immunostaining is slightly shrunken from the physiological brain, the boundary between 4+ and 4a- (0.5–0.6 mm lateral to the center of the paravermal vein) is likely to correspond to the physiological boundary between the A2 and C1 zones (i.e. 0.6 mm from the paravermal vein, see Methods). As our A2 recordings were located no more than 0.6 mm lateral from the paravermal vein (see Methods), we can therefore safely assume that the PCs we recorded in the A2 zone were all Z+.

The Z− band 4a− (∼0.18 mm wide, measured in crus IIb in the center panel of Fig. 9A) is located immediately lateral to 4+, followed by a very narrow Z+ band (4b+, ∼0.08 mm wide), a Z− band (4b−, ∼0.33 mm wide), a very narrow Z+ band (5a+, ∼0.05 mm wide) and a Z- band (5a−, ∼0.21 mm wide; making a total of 0.85 mm and Z+: Z− ratio of 0.13: 0.72). These measures were variable among lobules (crus IIa, IIb and paramedian lobule) and among individuals to some extent. As our C1 recordings were located within the range 0.6–1.3 mm lateral to the paravermal vein, we can assume that C1 zone mostly corresponds to the series of Z−/Z+ bands between 4a− and 5a− or the medial part of these series. Then, assuming a random positional sampling of PCs, we can estimate that a PC in the C1 zone in the areas of cortex under study has an 85% ( = 0.72/(0.13+0.72)) chance of being Z−.

Although the anatomical estimate indicates that random samples of PCs from the A2 and C1 zones from the lobules we recorded should be good surrogates for Z+ and Z− PC populations, respectively, it does not necessarily imply that the PCs in the actual dataset represent random samples. Thus, to test further whether the A2 and C1 data were Z+ and Z− surrogates, we generated mixed populations of Z+ and Z− PCs from the zebrin dataset by randomly sampling with replacement the Z+ and Z− identified PC populations. Each population had a total of 20 PCs. For each ratio of Z+ to Z− PCs (Z+/Z−: 20/0, 19/1, …, 0/20) 100 populations were generated, and the average value of a normalized spiking parameter (SS firing rate, SD and CV of the SS firing rate, and absolute pause duration) was calculated. The parameters were normalized such that the overall mean of each parameter was equal to one for the entire zonal and zebrin populations, which was done to eliminate the absolute differences in these parameters between the zonal and zebrin datasets. Thus, only the relative differences between Z+ and Z− cells, and correspondingly, A2 and C1 cells, remained.

The average value of each parameter from the simulated populations was then plotted as a function of the percentage of Z+ cells in the population (Fig. 9B, small red filled circles). The true experimental populations are indicated by large unfilled red circles at 0% (Z−) and 100% (Z+) on the plots, and closely match the averages of the corresponding simulated populations. A line was then fit (least squares) to the simulated data for each parameter. The parameter values for the A2 and C1 datasets were then entered into the linear regression equations to calculate an estimate of the percentage of Z+ cells in each zonal population.

Firing rate was the parameter showing the most robust difference between Z+ and Z− PCs and it provided estimates that most closely matched that of the anatomical estimates for the A2 (81.5% Z+) and C1 (−4.3% Z+, physically one cannot have less than 0% but the value of the zonal data falls slightly above that of the regression line at 0% leading to the negative percentage estimate) zones. Based on the four parameters the average estimate of the percentage of Z+ PCs for the C1 zone was 5.7±20.5%, and that for the A2 zone was 76.4±11.6%, both of which are close to that of the anatomical estimates.

In sum, both anatomical and statistical analyses suggest that our C1 zone recordings consisted of a pure or nearly pure Z− population of PCs. These analyses also support the use of our A2 zone recordings as a good, though not perfect, surrogate for a Z+ population because the large majority of the recordings are likely Z+.

## Discussion

The principal finding of this paper is that consistent differences exist in the spontaneous activity of PCs in different regions of the cerebellar cortex, which implies that the cerebellar cortex is functionally heterogeneous despite its uniform circuitry. In particular, SSs were found to have higher firing rates and less variable spike trains in Z− compared to Z+ bands. Significant differences in the basic interactions between SSs and CSs were also found. The PC recordings covered a significant amount of the cerebellar posterior lobe, suggesting that the observed differences in firing pattern between Z+ and Z− regions likely reflects a general organizational principle of the cerebellar cortex. Moreover, essentially the same findings were obtained for most of the parameters under two distinct types of anesthesia, indicating that the observed differences between the spontaneous firing patterns in Z+ and Z− bands are robust, and therefore that they likely will be observed in other physiological brain states.

### Methodological Issues

Most of the experiments were done under ketamine/xylazine anesthesia, which raises the issue of how applicable the results are to the awake brain state, in particular. The possibility that the observed differences in activity between Z+ and Z− PCs are specific to the ketamine anesthesia state was ruled out by finding similar differences using urethane anesthesia. Moreover, there are additional reasons to believe that the results have general validity. Specifically, the SS results appear to be robust in that similar differences between Z+ and Z− regions in SS firing rates and patterns, and in SS-CS interactions, were observed in two independent laboratories even though recordings were obtained from different rat strains using different (albeit similar) anesthetic and recording protocols, and the recorded PCs were located in different cerebellar regions. (Differences in CS firing rates were not consistent, but possible reasons for this are discussed later.) Furthermore, other aspects of cerebellar cortical physiology (e.g., CS synchrony patterns) are similar in ketamine/xylazine anesthetized preparations and in awake animals [54]–[59], suggesting that many of the parameters governing the cerebellar cortical network are similar in both the waking and ketamine-anesthetized conditions, though the specific activity patterns in the anesthetized animal almost certainly do not precisely match those that occur in the awake animal, and some parameters of activity can differ significantly [60]. Finally, while this paper was under review, similar findings were reported in awake mice, extending the generality of the findings of zebrin-related differences in spike activity [61]. In particular, the two studies found similar differences in SS firing rates and absolute SS pause duration between Z+ and Z− bands. Regarding CS activity Zhou et al. found higher rates in Z− cells whereas our datasets were not fully consistent.

### Possible mechanisms underlying differences in SS activity between Z+ and Z− bands

A number of mechanisms exist that potentially could explain the difference in SS firing rates between zebrin compartments observed here. The most straightforward is the possibility that there are systematic differences in the intrinsic excitability between PCs. Specifically, PCs have an intrinsic spike generator that allows them to fire spontaneously in the absence of synaptic drive [45], [62]–[64], and systematic variations in this generator between Z+ and Z− band PCs could lead to differences in SS firing patterns. Indeed, such a systematic regional variation has been described for the depolarization-induced slow current (DISC), which increases PC excitability, and which is expressed more strongly in posterior vermis PCs [65], [66], but posterior vermis is largely Z+, and thus DISC expression appears to be the opposite of what would explain the differences in SS rates found here. However, evidence has just been reported that differences related to the activity of TRPC3 (transient receptor potential cation channel type C3) in PCs contribute to differences in spiking characteristics between Z+ and Z− bands [61].

Alternatively, or in combination with any differences in PC intrinsic excitability, variations in the synaptic input to PCs could exist, either because of differences in the activity levels of the mossy fiber/granule cell input pathway to Z+ and Z− regions or because of differences in synaptically-related conductances between zebrin compartments. Differences in the activity levels of mossy fiber/granule cell input pathway to Z+ and Z− regions seems less plausible as a general mechanism, because the great diversity of mossy fiber sources makes it less likely for there to be a consistent matching of low and high activity levels to the Z+ and Z− bands, respectively. Also, while some mossy fiber systems align well with specific zebrin bands [8], [18], [24], in other cases the relationship is more complex [16].

Thus, a more likely possibility is that the synaptic efficacy of the parallel fiber-PC synapse varies between zebrin bands. Indeed, differences in the expression levels of several molecules exist between Z+ and Z− PCs that could lead to differences in synaptic transmission that could, in turn, explain the differences in SS rates we found. In particular, excitatory amino acid transporters (EAATs) act to limit EPSCs at parallel fiber synapses onto PCs [29]–[31]. One of these transporters, EAAT4, is found predominantly at perisynaptic regions on the PC dendritic membrane, and is highly expressed in Z+ PCs but not Z− PCs [25], [26], [67]. Given this differential expression of EAAT4, the excitatory drive from parallel fibers could be weaker in Z+ PCs, thereby leading to lower SS rates.

Another possible mechanism that could underlie the differences in overall SS firing rates is differential activation of molecular layer interneurons (MLIs; i.e., basket and stellate cells) in Z+ and Z− bands by parallel fiber activity [68]. Activation of a beam of parallel fibers leads to a traveling wave of excitation of the PCs and MLIs contacted by the parallel fibers [69], [70]. The activation of the MLIs then produces a wave of inhibition that travels in the wake of the excitatory wave to PCs directly underneath the parallel fiber beam, and to PCs located on either side of the parallel fiber beam [70], [71]. However, the amplitude of the evoked inhibition on PCs is not constant, but rather waxes and wanes as this second wave travels along the parallel fiber beam, such that strong activation of MLIs occurs in Z+ bands and weak activation in Z− bands [68]. Thus, parallel fiber drive to PCs would be more strongly countered by inhibition in the Z+ bands, which potentially would lead to lower SS firing rates.

The relative contributions of these and other possible mechanisms to the differences in SS activity we reported here will require study of synaptic currents and the intrinsic properties of PCs at the single and network level.

### Variations in CS firing rates between zebrin bands

We obtained mixed results when comparing CS activity across zebrin bands obtained from our different datasets. Nevertheless, that differences were found in two of three datasets suggests that CSs may also vary systematically between cerebellar cortical regions. However, multiple factors may underlie this variation, only some of which are correlated with zebrin expression. In fact, several factors that are known to contribute to CS firing rates can have opposite effects, and which one dominates may vary between different behavioral contexts, leading to a differing relationship between SS and CS activity levels.

For example, in the case of zonal recordings of CS firing rates, these were higher in the A2 zone, and thus varied inversely with SS rates. This inverse relationship is consistent with the well-known observation that changes in CS rates induced by direct manipulation of inferior olive excitability produce an inverse effect on SS rates [41]–[44], [72]. This inverse correlation of SS and CS is driven, at least in part, by climbing fiber activation of cerebellar cortical interneurons [43], [50], [73], [74] (however, see, [41], [42]), and is likely mediated via spillover of glutamate from climbing fiber-PC synapses [75]–[77].

In contrast, in the zebrin dataset a positive correlation of CS and SS firing rates was found across Z+ and Z− bands (i.e., both were higher in the Z− band). Such a relationship could be driven by the difference in SS activity that feeds into the disynaptic PC-cerebellar nucleo-inferior olivary pathway. Given the closed loop nature of the pathways interconnecting the cerebellar cortex, cerebellar nuclei and inferior olive, and that Z+ and Z− PCs project to separate cerebellar nuclear regions [23], [78], this raises the possibility that the relatively higher SS activity in Z− bands would more strongly inhibit the cerebellar nuclei. This, in turn, could lead to a relatively stronger disinhibition of the inferior olivary regions projecting back to the Z− bands, resulting in increased CS activity. Support for the significance of this mechanism comes from studies showing manipulation of SS levels in discrete cortical regions produces correlated changes in CS activity [46], [47].

During behavior, examples of both positive and negative correlation of SSs and CSs exist [79], [80]. In sum, the inconsistency with regard to CS firing rate we observed may be attributable to the fact that CSs are evoked by inferior olivary activity, which is not part of the cerebellar cortex. As a result CSs, are likely to be subject to more factors uncorrelated with the pattern of zebrin expression than is the case for the cerebellar cortical neurons responsible for generating and modulating SS activity.

### CS-SS interactions

Although the absolute duration of the pause in SSs following a CS was longer in Z+ bands, a greater active suppression of SS activity was found for Z− regions when SS firing rate differences were controlled for. Evidence indicates that the pause in SSs is at least partly caused by activation of MLIs and GABA release [46], [50], [73], [74]. However, MLIs appear not to receive climbing fiber synapses [81], but instead are activated by climbing fiber activity via glutamate spillover from climbing fiber-PC synapses [75], [76], [82]. EAAT4 is found in the perisynaptic membrane of climbing fiber-PC synapses [25], [67], and controls glutamate spillover at those synapses [27], [83], raising the possibility that the differing EAAT4 expression levels may also underlie the observed differences in active suppression of SSs between Z+ and Z− bands. That is, the relatively weak expression of EAAT4 in Z− band PCs could allow climbing fiber activity to cause greater MLI activation, leading to greater active inhibition of SSs following each CS.

The post-CS pause in SSs is generally followed by a subsequent modulation of SSs [53], which was found to be more pronounced in Z+ regions, although this effect was subtle. This difference may also be ascribable to variations in EAAT4 expression. This is because the post-pause SS modulation is also likely mediated by MLIs, as local block of GABA-A receptors reduces this modulation, for both negative and positive directions [46]. Moreover, glutamate spillover has recently been shown to cause not only activation of MLIs but also to lead to feed-forward inhibition of MLIs, which can last on the order of 100 ms [82], the same timescale as the duration of the modulation. Thus, in Z− bands the lower EAAT4 level allows the CS to activate more strongly the MLI network, which can then more strongly modulate SS activity.

### Implications for cerebellar function

The differences in spontaneous PC activity between Z+ and Z− bands observed here are raise the possibility that information arriving via different cerebellar afferent systems could be transformed by networks characterized by quite distinct operational states. Moreover, it may be more complex than a simple binary choice (Z+ versus Z−), because not all afferent systems respect the zebrin-defined compartments. In this regard it is interesting to compare the band and zone results, because while the bands and zones are closely aligned in the cortical regions we recorded, they are not congruent, and although the zebrin and zonal datasets showed the same trends (except for CS firing rates), the stronger, more statistically significant results were always found for the zebrin dataset. This suggests that the observed firing pattern differences are a reflection of the intrinsic organization of the cerebellar cortex, and that differences in the spiking patterns between zones are likely a secondary consequence of the close spatial alignment between zones and bands from the recorded regions. However, since the degree of alignment between bands and zones varies among afferent systems and cerebellar cortical regions, the Z+/Z− ratio of an afferent's target region will vary among afferents, and thus may be an important functional parameter for determining the type of information processing that different input signals undergo.

Finally, the difference in the regularity of the firing patterns between Z+ and Z− PCs may have important implications for the transmission of information from the cerebellar cortex to the cerebellar nuclei. This is because Z+ and Z− PCs target distinct regions in the cerebellar nuclei [23], [78]. PCs are often assumed to use rate coding, and if this is the case, the greater variability in the ISI distribution of Z+ PCs raises the possibility that information transmission from Z+ bands to the cerebellar nuclei is less efficient, as the efficiency of a rate code depends strongly on the regularity of the firing pattern [84].

The functional significance of this difference in SS regularity is difficult to gauge, because of uncertainties about the actual code used by PCs and about the exact convergence ratio of PCs to nuclear neurons. For example, a large degree of convergence in the PC-cerebellar nuclear projection, as suggested by anatomical results [85], could minimize the importance of differences in regularity by allowing averaging across the converging PC population, assuming that the “noise” in the SS activity in different PCs is independent. However, several physiological studies have suggested that the convergence in the PC-cerebellar nuclear projection may be relatively small (on the order of 10's:1 rather than 100's:1) [4], [86], in which case the differing noise levels may have a significant impact on coding efficiency. Regardless of what the exact convergence ratio turns out to be, if PCs really only use rate coding, it seems surprising to have approximately half of them generate significantly more irregular spike trains. Thus, the present results suggest that SSs use additional coding mechanisms, consistent with other reports (for review see [87].

Increased irregularity in SS activity has been proposed as a cause of motor coordination disorders including ataxia and dystonia [88]–[90]. However, recent theoretical and experimental findings have questioned the association of irregular SS firing patterns and motor coordination deficits [91], [92]. Our finding that PCs can display differing levels of firing regularity under normal physiological conditions would also argue against irregular firing patterns necessarily being pathological. Instead, as suggested above, this variation in regularity suggests that the cerebellar cortex employs multiple coding schemes, and that the specific code(s) used may vary between cerebellar regions. These results need to be verified in awake animals, but the demonstration of regularity differences in spiking between Z+ and Z− bands under two distinct anesthetic states raises the likelihood of this being an important characteristic of cerebellar activity that needs to be taken into account in future hypotheses of cerebellar function.
